# IRE1α: a gatekeeper of chemotherapy-induced immunogenicity in triple-negative breast cancer

**DOI:** 10.1038/s41392-025-02134-w

**Published:** 2025-02-12

**Authors:** Nirmal Robinson, Ruhi Polara, Daniel Thomas

**Affiliations:** 1 Centre for Cancer Biology, University of South Australia and SA Pathology, Adelaide, SA Australia; 2 Adelaide Medical School, Faculty of Health and Medical Sciences, The University of Adelaide, Adelaide, SA Australia; 3 Precision Cancer Medicine Theme, South Australian Health and Medical Research Institute (SAHMRI), Adelaide, SA Australia

**Keywords:** Breast cancer, Breast cancer

In a recent study published in *Cell*, Xu et al. have uncovered a critical role for the endoplasmic reticulum (ER) stress sensor inositol-requiring enzyme 1 alpha (IRE1α) in modulating the immunogenic effects of taxane chemotherapy in triple-negative breast cancer (TNBC). They reveal how IRE1α acts as a defense mechanism in cancer cells, preventing the accumulation of danger signals and subsequent immunogenic cell death (ICD).^1^

TNBC is an aggressive breast cancer subtype lacking estrogen receptor, progesterone receptor, and HER2 expression, which makes it unresponsive to targeted hormonal or HER2 therapies. Although breast cancer, particularly TNBC, has been historically considered a “cold” cancer in the context of immunotherapy, certain TNBC subtypes exhibit increased intra-tumoral lymphocyte infiltration and a higher mutation burden suggesting potentially higher antigen load. Increasingly immunotherapy such as anti-PD1 (pembrolizumab) is being added to taxane-based regimens prior to surgical removal for TNBC patients. Chemotherapy remains the main treatment option, yet TNBCs often develop resistance and escape immune detection. ICD, a form of tumor cell death triggered by certain chemotherapies, offers hope to enhance immune system activation against TNBC. However, the tumor’s ability to adapt to stress often limits effective ICD. Xu et al. identify that the ER stress sensor IRE1α is a pivotal regulator of chemotherapy-induced immunogenicity in TNBC, presenting an opportunity for therapeutic intervention.^1^

**Fig. 1:**
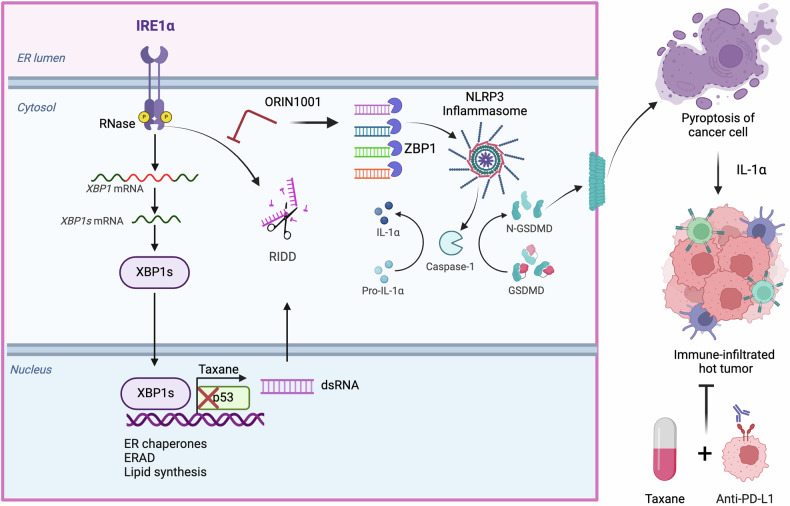
IRE1α‘s RNase activity, in addition to mitigating ER stress via its splicing function, silences double-stranded RNA (dsRNA) induced by taxane chemotherapy through regulated IRE1-dependent decay (RIDD). This suppression prevents activation of the NLRP3 inflammasome and subsequent pyroptosis, a highly immunogenic form of cell death. The tumor suppressor p53 represses the transcription of dsRNA. In p53-mutant triple-negative breast cancers (TNBCs), specific inhibition of IRE1α RNAase activity in combination with taxane chemotherapy promotes inflammasome activation, increases immune infiltration, and sensitizes tumors to immune checkpoint inhibitors (ICIs), such as anti-PD-L1. The figure was created using BioRender

ICD is characterized by the release of damage-associated molecular patterns, including calreticulin, extracellular ATP, and HMGB1. These molecules function as immunostimulatory signals that activate antigen-presenting cells (APCs) such as dendritic cells (DCs), bridging innate and adaptive immunity. While chemotherapeutic agents are known to induce ICD, the tumor’s intrinsic stress response mechanisms often counteract the full extent of this immunogenic effect.^2^ The study by Xu et al. reveals that IRE1α, a major component of the unfolded protein response, plays a dual role in TNBC’s response to chemotherapy. IRE1α activation, triggered by chemotherapy-induced ER stress, initiates the splicing of XBP1 mRNA (XBP1s), a transcription factor that restores ER homeostasis and promotes cell survival under stress conditions. Interestingly, this adaptive response not only supports TNBC cell viability but also dampens the immunogenicity of chemotherapy such as taxane. Mechanistically, IRE1α activation silences double-stranded RNA (dsRNA) released because of taxane-induced DNA damage through a process called IRE1-dependent decay, which also contributes to tumor suppression by degrading specific microRNAs that repress apoptosis and mRNA of genes that regulate lipid metabolism.^3^ Specific inhibition of IRE1α RNase activity or its loss leads to accumulation of dsRNA, which enhances NLRP3 inflammasome activation-mediated ICD known as pyroptosis (Fig. 1). Moreover, p53 provides another layer of protection as it preferentially binds to the promoters and enhancers of dsRNA-prone transcripts and repress the expression of dsRNA induced upon taxane treatment. It would be interesting to know if sXBP1 could have a similar function or if it cooperates with p53.

The mechanisms that enhance immunogenicity during chemotherapy are not well understood. Xu et al. describe IRE1α as a critical factor that dampens the immunostimulatory effects of taxane, thus preventing the innate immune recognition in cold tumors such as TNBC. Pyroptosis is a form of programmed inflammatory cell death that has been implicated in several cancers. It is considered a double-edged sword with respect to cancers, as long-term exposure to an inflammatory environment can either promote or prevent tumorigenesis.^4^ ORIN1001, a pharmacological inhibitor of IRE1α, specifically inhibits the RNase activity without affecting its splicing function. Currently, ORIN1001 is undergoing clinical evaluation for its safety and efficacy in treating advanced solid tumors. Inhibition of IRE1α in p53-deficient immunologically cold TNBC allows taxane treatment to induce extensive dsRNA accumulation, which is sensed by ZBP1, which in turn activates NLRP3 inflammasome but is independent of sensing by RIG-I-MAVS pathway. NLRP3 inflammasome activation results in Gasdermin D (GSDMD)-mediated pyroptosis of TNBC cells. Gasdermins are a family of pore-forming proteins that play a central role in pyroptosis. They are cleaved to their active forms by Caspase-1/3/8/11 in a context-dependent manner.^4^ Pyroptosis is specifically regulated by GSDMD activated by Caspase 1, but independent of GSDME and other caspases in IRE-1α-inhibited, taxane-treated cells. The authors note that inflammasome activation is specific to TNBC cells due to increased secretion of interleukin (IL)-1α rather than IL-1b, which is mostly secreted by myeloid cells, such as macrophages. Furthermore, pyroptosis was distinctly observed when ORIN1001 was combined with taxane and not in combination with other chemotherapeutic agents, including carboplatin, doxorubicin, or cyclophosphamide.

DCs and macrophages are APCs critical for initiating adaptive immune responses. Their interaction with pyroptotic cancer cells can influence the outcome of tumor immunity. Consistent with a recent report in prostate cancer,^5^ single-cell RNA sequencing has revealed that IRE1α-inhibited TNBC cells treated with chemotherapy markedly increased the inflammatory M1-like macrophages and priming of DCs as evidenced by increased expression of major histocompatibility complex (MHC)-II and costimulatory molecules like CD80 and CD86. This could expose tumor antigens to DCs, which process and present these antigens on MHC-I molecules to activate CD8+ cytotoxic T lymphocytes. Consistently, infiltration of CD8+ T cells inversely correlated with IRE1α RNAase activity. The study demonstrates that combining an IRE1α RNase inhibitor with taxane chemotherapy transforms cold breast tumor microenvironment (TME) into an inflamed TME. ORIN1001 also can convert programmed death ligand 1 (PD-L1)-negative, immune checkpoint inhibitor (ICI)-unresponsive TNBC tumors into PD-L1 high immunogenic tumors that are hypersensitive to ICI therapy.

This study positions IRE1α as a critical regulator of tumor–immune interactions in TNBC and highlights the potential of targeting IRE1α as a novel strategy to overcome immunosuppressive mechanisms in TNBC. By suppressing IRE1α activity, chemotherapy-induced ER stress can be leveraged to maximize ICD and elicit robust anti-tumor immune responses. This approach may be particularly effective in combination with immune checkpoint and indoleamine 2,3-dioxygenase (IDO1) inhibitors, offering a promising avenue for enhancing immunotherapy outcomes in TNBC patients, offering a new therapeutic target to enhance chemotherapy-induced immunogenicity, potentially transforming the treatment landscape for TNBC.

The applicability of the findings to all TNBC patients remains uncertain due to the heterogeneity of the disease, necessitating further investigation. The study highlights that the effects of IRE1α inhibition are specific to taxane-based chemotherapy. However, the underlying mechanisms driving differential immune responses to various chemotherapies remain poorly understood and warrant deeper exploration. Furthermore, while the preclinical findings are promising, they must be validated through clinical trials to assess the safety and efficacy of IRE1α inhibitors in combination with taxanes in TNBC patients. Lastly, the study does not address the long-term effects of IRE1α inhibition on tumor recurrence and overall patient survival, underscoring the need for continued research in these areas.
